# Control of solvent production by sigma‐54 factor and the transcriptional activator AdhR in *Clostridium beijerinckii*

**DOI:** 10.1111/1751-7915.13505

**Published:** 2019-11-06

**Authors:** Bin Yang, Xiaoqun Nie, Yang Gu, Weihong Jiang, Chen Yang

**Affiliations:** ^1^ CAS‐Key Laboratory of Synthetic Biology CAS Center for Excellence in Molecular Plant Sciences Shanghai Institute of Plant Physiology and Ecology Chinese Academy of Sciences Shanghai China; ^2^ University of Chinese Academy of Sciences Beijing China

## Abstract

Clostridia are obligate anaerobic bacteria that can produce solvents such as acetone, butanol and ethanol. Alcohol dehydrogenases (ADHs) play a key role in solvent production; however, their regulatory mechanisms remain largely unknown. In this study, we characterized the regulatory mechanisms of two ADH‐encoding genes in *C. beijerinckii*. SigL (sigma factor σ^54^) was found to be required for transcription of *adhA1* and *adhA2* genes. Moreover, a novel transcriptional activator AdhR was identified, which binds to the σ^54^ promoter and activates σ^54^‐dependent transcription of *adhA1* and *adhA2*. *Clostridia beijerinckii* mutants deficient in SigL or AdhR showed severely impaired butanol and ethanol production as well as altered acetone and butyrate synthesis. Overexpression of SigL resulted in significantly improved solvent production by *C. beijerinckii* when butyrate was added to cultures. Our results reveal SigL as a novel engineering target for improving solvent production by *C. beijerinckii* and other solvent‐producing clostridia. Moreover, this study gains an insight into regulation of alcohol metabolism in diverse clostridia.

## Graphical abstract

A novel regulatory system of alcohol synthesis in *Clostridia beijerinckii* was characterized. This study provides a new insight into regulation of ADH‐encoding genes in diverse Clostridium species and a novel engineering target to improve solvent production in solvent‐producing clostridia.

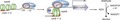

## Introduction

The clostridial solvent production, also known as the acetone–butanol–ethanol (ABE) fermentation, was one of the largest industrial fermentation processes early in the 20th century (Durre, 2007). Due to the growing concerns on energy security and climate change, biological production of butanol has been receiving renewed interest during the last 10 years (Lee *et al.*, 2008; Papoutsakis, 2008; Cheng *et al.*, 2019). This is because butanol possesses superior fuel properties compared with ethanol (Durre, 2007; Lee *et al.*, 2008). Clostridia, which are Gram‐positive obligate anaerobes, can produce fuels and chemicals through fermentation of a variety of carbon sources including sugars, cellulose, and CO_2_ or CO (Durre, 2007; Tracy *et al.*, 2012; Ren *et al.*, 2016). Among solventogenic clostridia, *Clostridium beijerinckii* is one of the best‐studied species (Lee *et al.*, 2008; Ezeji *et al.*, 2010). Random mutagenesis has been used to enhance its solvent production (Green, 2011). Attempts have also been made to improve the strain performance based on genetic engineering strategies (Charubin *et al.*, 2018; Cheng *et al.*, 2019).

Alcohol dehydrogenases (ADHs) play a key role in solvent production in clostridia, which catalyze the interconversion between aldehydes and ketones and their corresponding alcohols (Reid and Fewson, 1994; Radianingtyas and Wright, 2003). A few global transcriptional regulators including Spo0A, CcpA, AbrB and Rex have been found to regulate ADH‐encoding genes in *Clostridium acetobutylicum* (Yang *et al.*, 2018). However, little is known about the regulatory mechanisms of ADH‐encoding genes in other clostridia such as *C. beijerinckii*. Among the 20 ADH‐encoding genes in *C. beijerinckii*, two genes (Cbei_2181 and Cbei_1722) encoding iron‐containing/activated ADHs were strongly induced at the onset of solvent production, suggesting that they are responsible for synthesis of butanol and ethanol (Wang, *et al.*, 2012). Unlike in *C. acetobutylicum*, lack of the redox‐sensing regulator Rex did not affect the fermentation product formation in *C. beijerinckii*, suggesting that this species may have another mechanism for modulating alcohol production Zhang *et al.*, 2014). Recently, sigma factor σ^54^ was found to play a central role in carbon metabolism in *C. beijerinckii *(Hocq *et al.*, 2019), but the exact regulatory mechanism remains to be elucidated.

σ^54^ is unique in that it shares no detectable homology with any of the other known sigma factors and binds to conserved −12 and −24 promoter elements (Buck and Cannon, 1992; Barrios *et al.*, 1999). The σ^54^‐dependent transcription absolutely requires the presence of an activator that couples the energy generated from ATP hydrolysis to the isomerization of the RNA polymerase‐σ^54^ closed complex (Schumacher *et al.*, 2004; Bush and Dixon, 2012). These activators, also called enhancer‐binding proteins (EBPs), bind to upstream activator sequences (UAS) located upstream of the promoter. In a previous study, we have identified putative EBPs and reconstructed σ^54^ regulons in diverse *Clostridium* species by using comparative genomic approaches (Nie *et al.*, 2019). The reconstructed σ^54^ regulons contain the genes associated with butyrate and alcohol synthesis. A large number of ADH‐encoding genes were predicted to be regulated by σ^54^ (SigL) and individual EBPs (Nie *et al.*, 2019).

In this study, we verified the putative σ^54^ promoter elements upstream of ADH‐encoding genes in the genomes of *Clostridium* spp. by using *in vitro* binding assays. We found that SigL is required for transcription of the *adhA1* (Cbei_2181) and *adhA2* (Cbei_1722) genes in *C. beijerinckii*. Then, a novel EBP, namely AdhR, was experimentally characterized as an activator of σ^54^‐dependent transcription of *adhA1* and *adhA2*. Furthermore, we studied the effects of disruption of SigL‐ or AdhR‐encoding genes on fermentation product formation. Our results indicated that SigL and AdhR control alcohol synthesis in *C. beijerinckii* and overexpression of SigL can improve the solvent production.

## Results

### SigL binds to the −12 and −24 promoter elements *in vitro*

**Figure 1:**
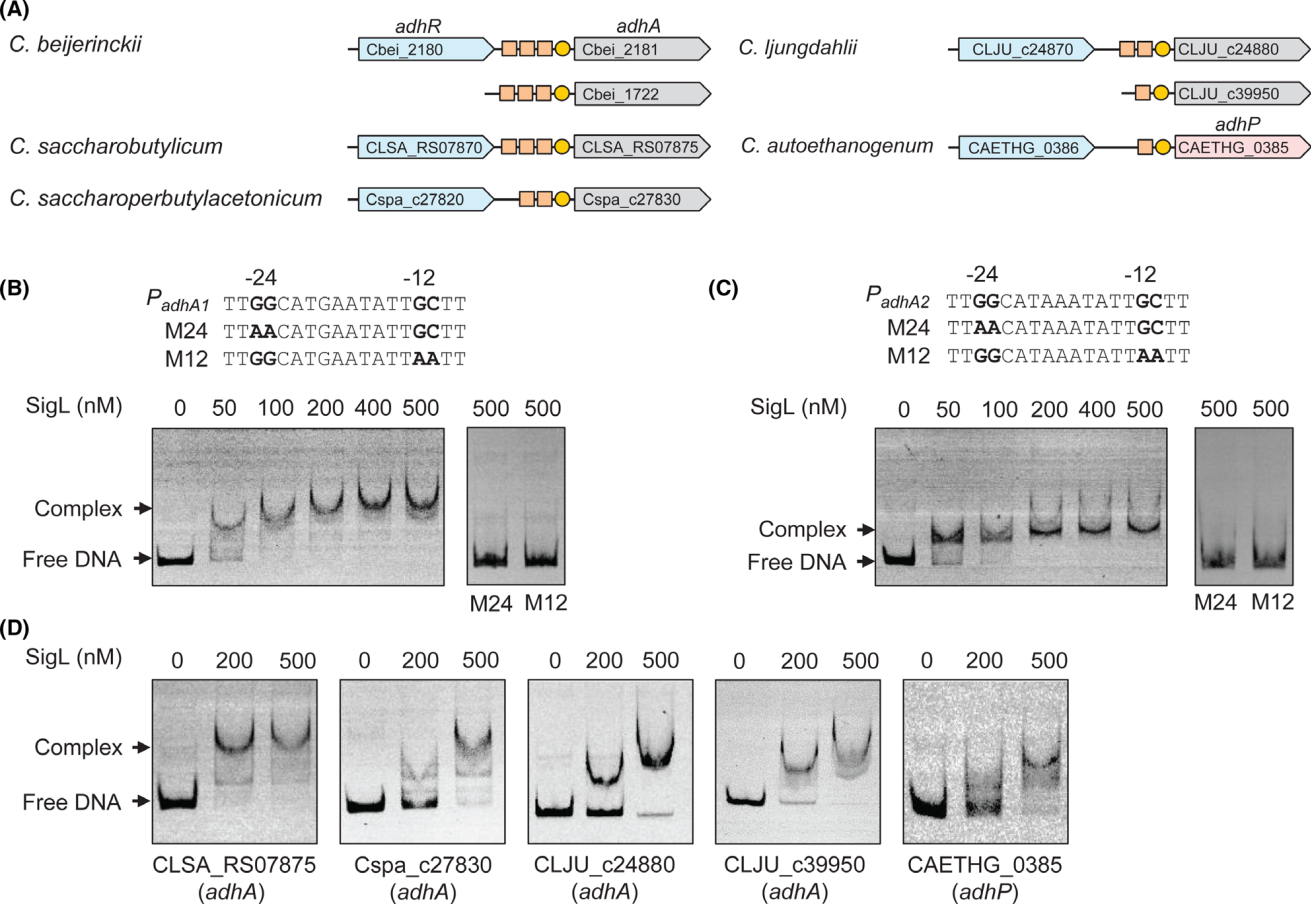
SigL binds to −12 and −24 promoter elements upstream of ADH‐encoding genes. A. Genomic context of ADH‐encoding genes in *Clostridium* species. Genes are shown by arrows. Predicted binding sites of SigL and AdhR are shown by yellow circles and squares respectively. B, C. EMSAs with purified SigL protein and DNA fragments from the promoter region of *adhA1* (B) and *adhA2* (C). Binding of SigL to the promoter fragments with mutations at −24 (M24) and −12 (M12) elements was also shown. D. EMSAs with purified SigL protein and DNA fragments from the promoter regions of ADH‐encoding genes in *Clostridium* species other than *C. beijerinckii*. The DNA fragments contain the putative −12 and −24 elements.

To validate the predicted regulation of clostridial ADH‐encoding genes by σ^54^ (Fig. 1A), electrophoretic mobility shift assays (EMSAs) were performed using the recombinant SigL protein from *C*. *beijerinckii*, which was overexpressed in *E. coli* with the *N*‐terminal His_6_ tag and purified with a nickel‐chelating affinity column. The DNA fragments from the promoter regions of *adhA1* and *adhA2* in *C*. *beijerinckii*, which contain the putative −12 and −24 promoter elements, were tested in EMSAs. We observed that SigL protein binds to the DNA fragments in a concentration‐dependent manner (Fig. 1B and C). The DNA fragments were completely shifted with 200 nM SigL. In contrast, no binding was observed for the mutated fragments with substitutions of the strictly conserved GC and GG dinucleotides at −12 and −24 regions, respectively, even at 500 nM SigL protein (Fig. 1B and C). Thus, SigL binds to the −12 and −24 promoter elements of *adhA1* and *adhA2* in *C*. *beijerinckii*.

Electrophoretic mobility shift assays were also performed to assess the predicted SigL‐binding sites upstream of the ADH‐encoding genes in other clostridia. Conserved −12 and −24 elements were identified in the promoter region of 17 *adhA* genes encoding iron‐containing/activated ADHs and of 20 *adhP* genes encoding zinc‐dependent ADHs in our previous study (Nie *et al.*, 2019). Among them, five DNA fragments were amplified from the promoter regions of *adhA* or *adhP* genes in *Clostridium saccharobutylicum*, *Clostridium saccharoperbutylacetonicum*, *Clostridium ljungdahlii* and *Clostridium autoethanogenum* (Fig. 1A). These DNA fragments were tested for binding of *C. beijerinckii* SigL protein that is well conserved in clostridia. A shift in the presence of purified SigL was observed for all the five fragments (Fig. 1D), suggesting that regulation of the ADH‐encoding genes by SigL is conserved in diverse *Clostridium* species.

### SigL is required for transcription of *adhA1* and *adhA2* genes in *C. beijerinckii*

**Figure 2:**
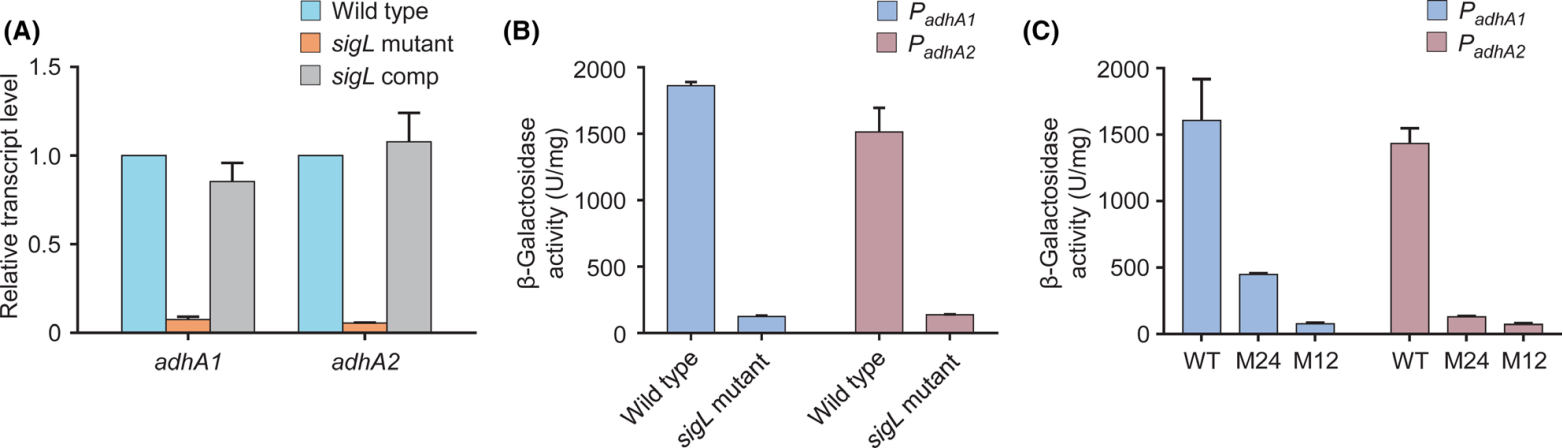
SigL is required for transcription of *adhA1* and *adhA2* genes. A. Effect of *sigL* inactivation on transcript levels of *adhA1* and *adhA2*. The transcript levels of each gene in the *sigL*‐inactivated mutant (*sigL* mutant) and *sigL*‐complemented strain (*sigL* comp) were normalized to the gene expression in the wild type. B. Effect of *sigL* inactivation on expression from *adhA1* and *adhA2* promoters. The activities of *adhA1* and *adhA2* promoters were determined using β‐galactosidase assays. C. Effect of mutations of SigL‐binding sites on *adhA1* and *adhA2* promoter activities. See Fig. 1 for M24 and M12. *Error bars* represent the standard deviation of three biological replicates.

To examine whether SigL regulates expression of *adhA1* and *adhA2* genes, the *sigL* gene (Cbei_0595) in *C. beijerinckii* was disrupted by inserting an intron (Fig. S1). We compared the gene expression between the *sigL*‐inactivated mutant (*sigL* mutant) and the wild type by using quantitative real‐time PCR (qRT‐PCR). The transcript levels of both *adhA1* and *adhA2* were decreased by more than 90% in the *sigL* mutant compared with the wild type (Fig. 2A). Complementation of the *sigL* mutant by using a plasmid construct constitutively expressing *sigL* restored the transcript levels of *adhA1* and *adhA2*. We constructed *C. beijerinckii* strains expressing *lacZ* under the control of *adhA1* or *adhA2* promoters. The *sigL* mutant, compared with the wild type, exhibited markedly lower levels of expression from *adhA1* and *adhA2* promoters, as determined by β‐galactosidase assays (Fig. 2B). Moreover, mutations of the −12 and −24 elements for SigL binding decreased the activities of *adhA1* and *adhA2* promoters by 72−95% (Fig. 2C). These results indicate that SigL is required for transcription of *adhA1* and *adhA2* genes in *C. beijerinckii*.

### AdhR activates σ^54^‐dependent transcription of *adhA1* and *adhA2*

**Figure 3:**
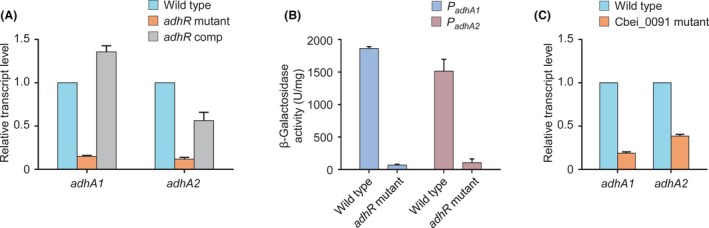
Effect of *adhR* inactivation on transcription of *adhA1* and *adhA2* genes. A. Effect of *adhR* inactivation on transcript levels of *adhA1* and *adhA2*. The transcript levels of each gene in the *adhR*‐inactivated mutant (*adhR* mutant) and *adhR*‐complemented strain (*adhR* comp) were normalized to the gene expression in the wild type. B. Effect of *adhR* inactivation on expression from promoters of *adhA1* and *adhA2*. C. Effect of inactivation of the gene encoding a small heterodimeric protein HU (Cbei_0091) on transcript levels of *adhA1* and *adhA2*. The transcript levels of each gene were normalized to the gene expression in the wild‐type strain. *Error bars* represent the standard deviation of three biological replicates.

In a previous study, we made the hypothesis that *adhA1* and *adhA2* genes are regulated by a novel EBP, namely AdhR, in *C. beijerinckii* (Nie *et al.*, 2019). To test it, we investigated the effect of inactivation of *adhR* (Cbei_2180) on target gene expression by using qRT‐PCR and β‐galactosidase assays. We observed that the transcript levels of *adhA1* and *adhA2* were decreased by 90% in the *adhR* mutant compared with the wild type (Fig. 3A). Consistently, compared with the wild type, the *adhR* mutant showed substantially lower levels of expression from *adhA1* and *adhA2* promoters (Fig. 3B). Complementation of the *adhR* mutant by using a plasmid expressing *adhR* increased the expression of *adhA1* and *adhA2* (Fig. 3A). Thus, AdhR is a positive regulator of *adhA1* and *adhA2* genes. AdhR contains a central AAA^+^ (ATPase associated with various cellular activities) domain that is highly homologous (> 50% sequence identity) to the σ^54^‐interacting domain of transcriptional activators NtrC and NifA (Martinez‐Argudo *et al.*, 2004; De Carlo *et al.*, 2006). We found that a small histone‐like protein HU (encoded by Cbei_0091), which shares sequence homology with the HBsu protein from *B. subtilis* (Micka and Marahiel, 1992), was also involved in transcription of *adhA1* and *adhA2* (Fig. 3C). The HU protein may facilitate DNA looping to enable the interaction between σ^54^ and AdhR (Hoover *et al.*, 1990).

**Figure 4:**
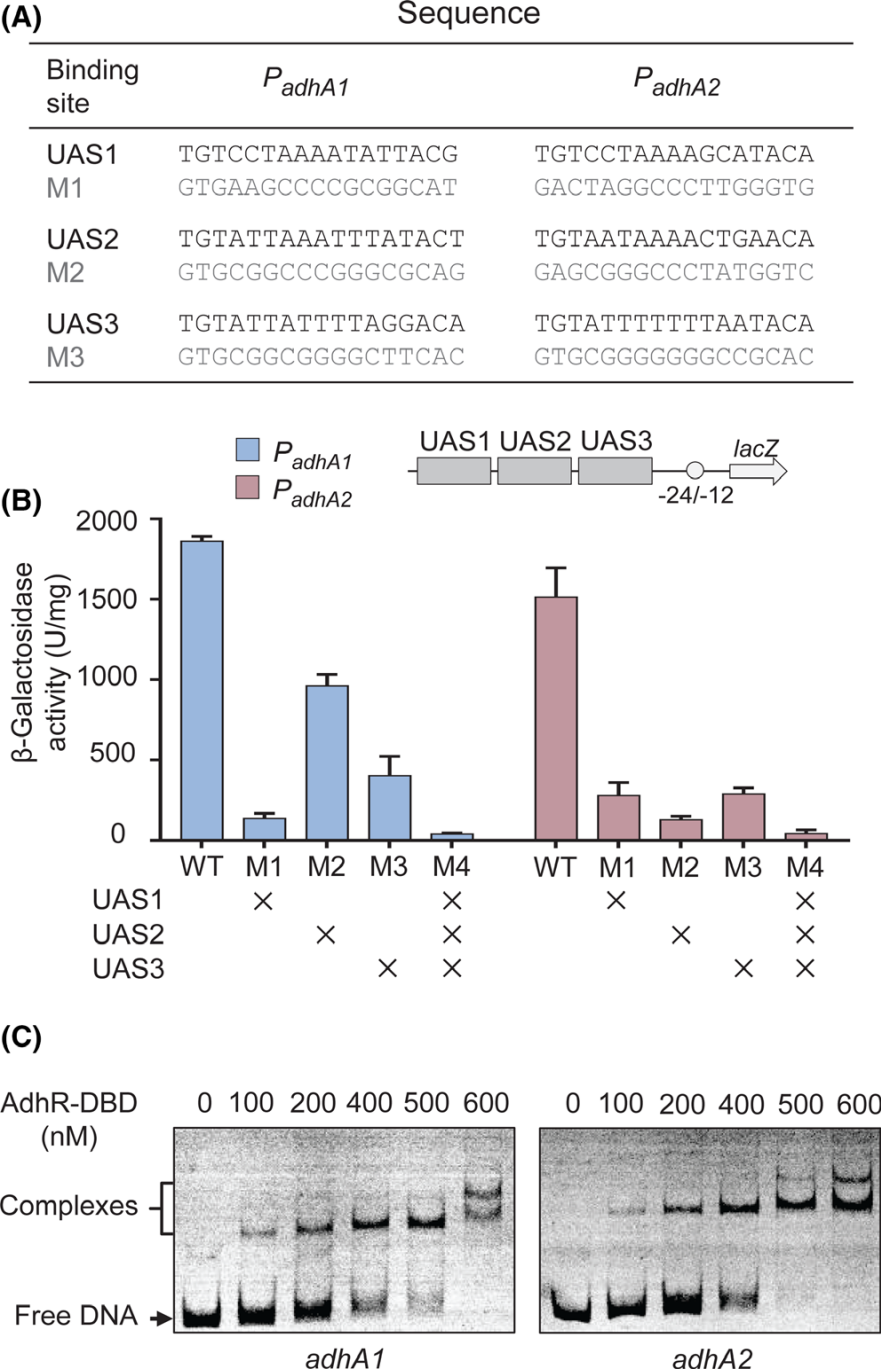
AdhR activates *adhA1* and *adhA2* promoters by binding to three UAS sites. A. Mutations introduced into each of the three UAS sites (M1 − M3) in the *adhA1* and *adhA2* promoters. The mutated sequences are shown below the wild‐type sequences. B. Effect of UAS mutations on activities of *adhA1* and *adhA2* promoters. The mutations are indicated by ‘×’. *Error bars* represent the standard deviation of three biological replicates. C. EMSAs with purified *C. beijerinckii* AdhR‐DBD and DNA fragment from the promoter region of *adhA1* and *adhA2*.

The C‐terminal domain of AdhR protein is a helix–turn–helix (HTH) DNA‐binding domain (DBD). Three AdhR‐binding sites (i.e. UAS sites) upstream of the σ^54^ promoter elements was predicted for both *adhA1* and *adhA2* genes in our previous study (Nie, *et al.*, 2019). To test whether these UAS sites are involved in expression of *adhA1* and *adhA2*, mutations were introduced into individual UAS (Fig. 4A). We observed that disruption of any one of the three UAS sites severely impaired the activities of both *adhA1* and *adhA2* promoters and the triple mutation resulted in < 2% of the promoter activities (Fig. 4B). Thus, all the three UAS sites contribute to transcriptional activation of *adhA1* and *adhA2*. To confirm the direct binding of AdhR to the UAS sites, EMSAs were performed using purified recombinant DBD of AdhR protein (AdhR‐DBD) from *C. beijerinckii* and the DNA fragments containing all three UAS sites. For both *adhA1* and *adhA2* promoter fragments, shifted bands were visible in the presence of increasing amounts of AdhR‐DBD protein (Fig. 4C). Therefore, the above results indicate that AdhR binds to the three UAS sites upstream of the promoter and activates the transcription of *adhA1* and *adhA2* genes in *C. beijerinckii*.

### SigL and AdhR control solvent synthesis

**Figure 5:**
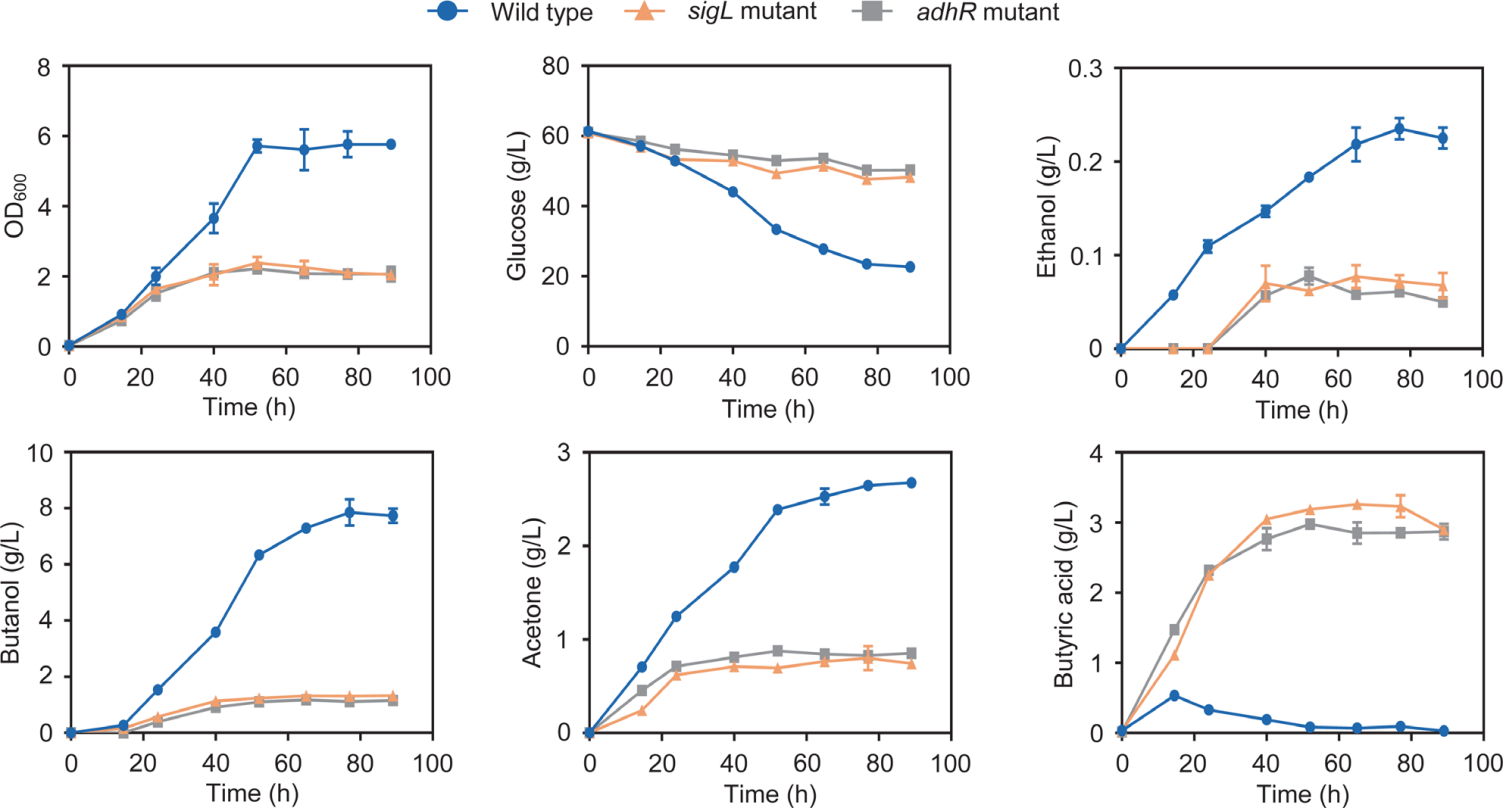
Cell growth and fermentation product formation in batch cultures of *C. beijerinckii* wild‐type strain and *adhR* and *sigL* mutants. The OD_600_, concentrations of glucose, ethanol, butanol, acetone and butyric acid in the medium were measured during the cultivation. *Error bars* represent the standard deviation of three biological replicates.

To elucidate the role of σ^54^ and AdhR in regulation of central metabolism in *C. beijerinckii*, we compared the cell growth and fermentation products between wild type and *adhR* and *sigL* mutants. Inactivation of *adhR* or *sigL* severely impaired the growth of the resulting strains on glucose (Fig. 5). Compared with the wild type, ethanol, butanol and acetone production by both *sigL* and *adhR* mutants decreased by 74%, 85% and 70% respectively (Fig. 5). Both mutants accumulated a high level (~ 3 g l^−1^) of butyric acid (Fig. 5). The production of acetic acid by the mutants was not changed significantly compared with other fermentation products. These results indicate that σ^54^ and AdhR not only control the butanol and ethanol production by directly regulating *adhA1* and *adhA2* genes but also influence the synthesis of acetone and butyric acid.

### Overexpression of SigL can improve solvent production by *C. beijerinckii*

**Figure 6:**
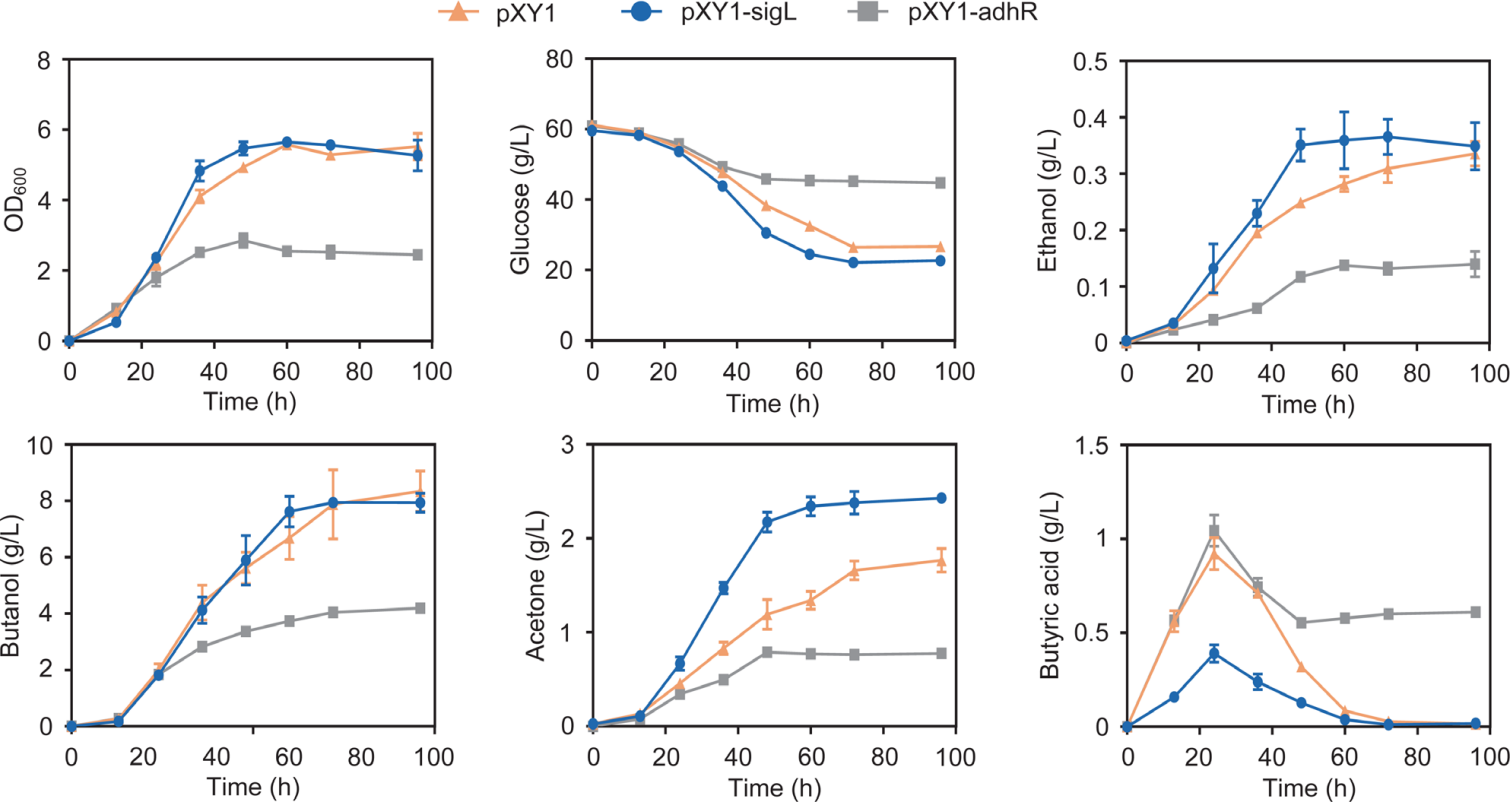
Cell growth and fermentation product formation in batch cultures of *C. beijerinckii* SigL‐ or AdhR‐overexpressing strains. The strain carrying an empty‐vector plasmid (pXY1) was used as a control. The OD_600_, concentrations of glucose, ethanol, butanol, acetone and butyric acid in the medium were measured during the cultivation. *Error bars* represent the standard deviation of three biological replicates.

To investigate the effect of overexpression of SigL and AdhR, we constructed *C. beijerinckii* strains, in which *sigL* or *adhR* genes from *C. beijerinckii* were expressed in a plasmid by the promoter of thiolase (*thl*). The strain carrying an empty‐vector plasmid was used as a control. Then, we compared the fermentation performances of the SigL‐ or AdhR‐overexpressing strains and the control strain in batch cultures. During the exponential growth phase, the SigL‐overexpressing strain exhibited slightly increased rates of glucose consumption and ethanol production compared to the control strain (Fig. 6). Although butanol production was almost unchanged by SigL overexpression, acetone production was greatly enhanced throughout the cultivation, leading to a 37% increase in the titre of acetone (Fig. 6). However, overexpression of AdhR resulted in decreases in cell growth and solvent production (Fig. 6). Interestingly, we observed that SigL overexpression led to drastically reduced synthesis and reassimilation of butyric acid (Fig. 6). This result suggests that the availability of butyryl coenzyme A (butyryl‐CoA) is limited in the SigL‐overexpressing strain (Tummala *et al.*, 2003; Sillers *et al.*, 2009).

**Figure 7:**
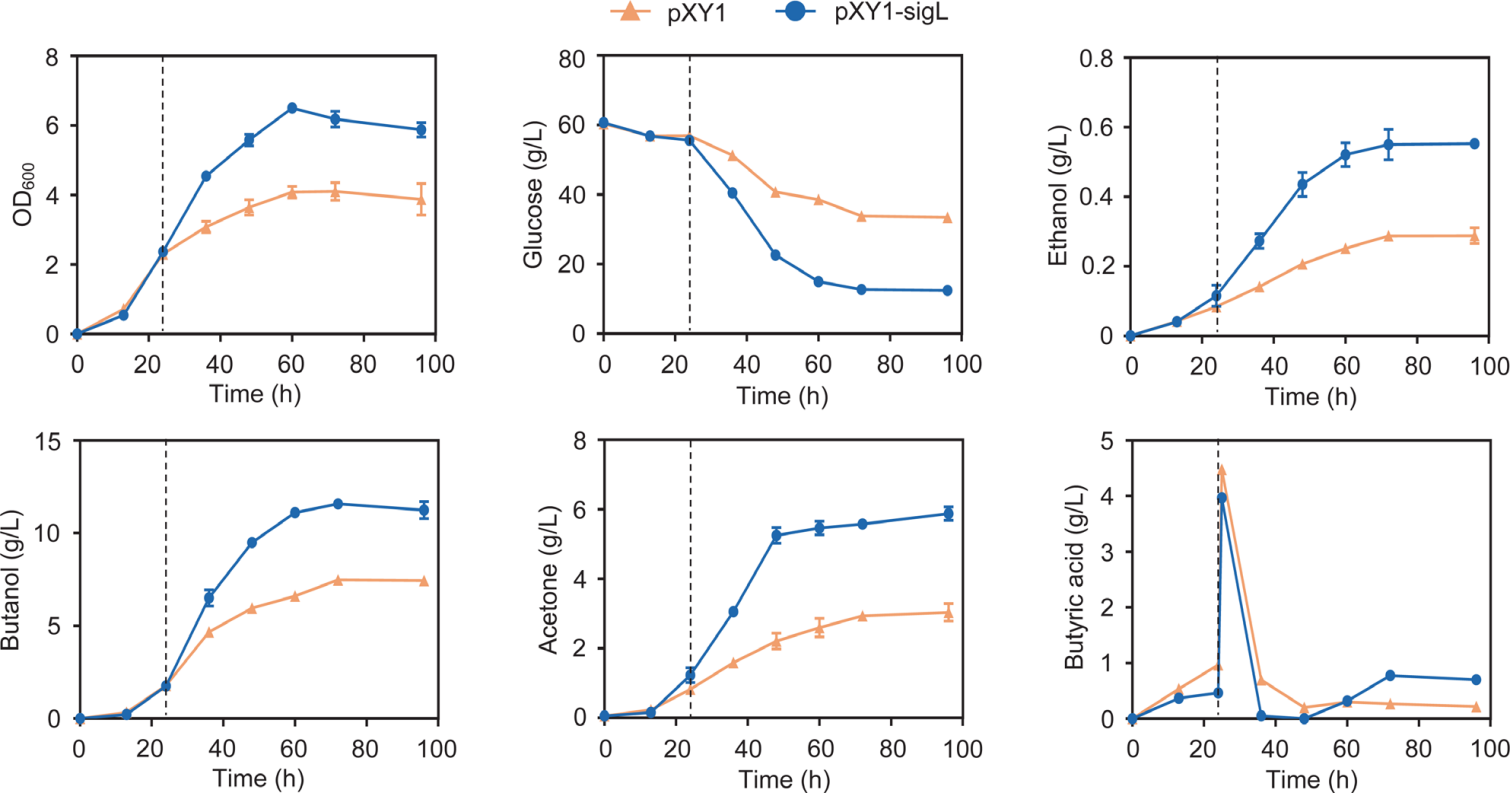
Product profiles of *C. beijerinckii* SigL‐overexpressing strain fermentation with addition of butyrate. The strain carrying an empty‐vector plasmid (pXY1) was used as a control. The vertical dashed line indicates the time at which butyrate was added to both the control strain and SigL‐overexpressing strain cultures. The OD_600_, concentrations of glucose, ethanol, butanol, acetone and butyric acid in the medium were measured during the cultivation. *Error bars* represent the standard deviation of three biological replicates.

To study whether an increased availability of butyryl‐CoA can improve the butanol production by the SigL‐overexpressing strain, we added 3.5 g l^−1^ of butyrate to the culture medium when the butyrate concentration started to decline. Exogenously added butyrate was quickly consumed by the SigL‐overexpressing strain, leading to a marked increase in butanol production from 7.9 to 11.25 g l^−1^ (Fig. 7). Compared with the control strain, the SigL‐overexpressing strain showed significantly increased rates of cell growth and glucose consumption after butyrate was added (Fig. 7). Production of butanol, ethanol and acetone was increased by 50%, 96% and 93%, respectively, by SigL overexpression (Fig. 7). The titre of total solvents was increased 1.6‐fold from 10.7 to 17.6 g l^−1^ in the SigL‐overexpressing strain compared to that in the control strain. By adding butyrate at the beginning of batch culture, we also observed enhanced solvent production by the SigL‐overexpressing strain compared with the control strain. Therefore, overexpression of SigL can significantly improve solvent production by *C. beijerinckii*.

## Discussion

**Figure 8:**
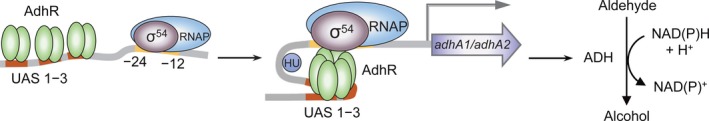
Schematic of the proposed mechanism for regulation of *adhA1* and *adhA2* genes by σ^54^ (SigL) and AdhR. σ^54^ directs the RNA polymerase holoenzyme to bind at the −12 and −24 promoter elements, and AdhR binds to three UAS sites upstream of the promoter. AdhR interacts with σ^54^ via DNA looping facilitated by HU protein and activates the σ^54^‐dependent transcription of *adhA1* and *adhA2* encoding ADHs, which catalyzes the conversion of aldehydes to alcohols.

In this study, SigL (σ^54^) was identified as a master regulator of solvent synthesis in *C. beijerinckii*. We found that SigL, which binds to −12 and −24 promoter elements, is required for transcription of *adhA1* and *adhA2* in *C. beijerinckii* (Fig. 8). Moreover, we identified AdhR as an activator of σ^54^‐dependent transcription of *adhA1* and *adhA2*. AdhR binds to three UAS sites upstream of the promoter. *C. beijerinckii* mutants deficient in SigL or AdhR showed severely impaired butanol and ethanol synthesis. We propose that AdhR interacts with σ^54^ via DNA looping facilitated by HU protein and activates the σ^54^‐dependent transcription of *adhA1* and *adhA2*, leading to ADH‐catalyzed conversion of aldehydes to alcohols (Fig. 8). Based on the understanding of regulation of alcohol synthesis, we overexpressed SigL in *C. beijerinckii*, which resulted in significantly improved solvent production.

Genetic engineering has been applied to enhance solvent production by *C. beijerinckii*; however, most of the attempts did not result in a desired solvent producer (Lu *et al.*, 2017; Wen *et al.*, 2017). Although a few transcriptional factors including Spo0A, CcpA, AbrB and Rex have been found to regulate solvent production in *C. acetobutylicum* (Yang *et al.*, 2018), little is known about the regulatory mechanisms of solvent production in other clostridia. Our finding, which indicates that alcohol synthesis is controlled by σ^54^, proposes a novel engineering target for improving solvent production by *C. beijerinckii* and other solvent‐producing clostridia. As expected, SigL overexpression resulted in increased ethanol synthesis by directly upregulating *adhA1* and *adhA2* in *C. beijerinckii*. Butanol production was also significantly enhanced by SigL overexpression when butyryl‐CoA availability was increased by adding butyrate to cultures. The SigL‐overexpressing strain also exhibited a remarkable increase in acetone production compared to the control strain. A previous report has shown that overexpression of the gene for alcohol synthesis led to upregulation of the genes involved in acetone synthesis (*ctfA*, *ctfB* and *adc*) in *C. acetobutylicum* (Tummala *et al.*, 2003). Thus, we speculate that SigL may indirectly modulate acetone synthesis via upregulation of *adhA1* and *adhA2*. In addition, the increased glucose consumption rate of the SigL‐overexpressing strain may be explained by enhancement of glycolytic activity by decreased NADH/NAD^+^ ratio due to an increase in alcohol production (Liang *et al.*, 2013). The solvent production by the SigL‐overexpressing strain could be further improved by engineering cellular metabolism for the increased availability of butyryl‐CoA as well as optimizing fermentation and product recovery processes (e.g. using gas stripping for solvent recovery) (Ezeji *et al.*, 2003; Hou *et al.*, 2013).

AdhR is a novel activator of σ^54^‐dependent transcription. The C‐terminal domain of AdhR directs its binding to UAS sites, and the central AAA^+^ domain is responsible for interaction with σ^54^. In addition, AdhR contains an N‐terminal GAF domain (named for cyclic GMP‐specific and stimulated phosphodiesterases, *Anabaena* adenylate cyclases and *E. coli* FhlA) and a PAS (Per, ARNT and Sim) domain, which are assumed to have a role in signal perception, and modulates the activity of AdhR (Aravind and Ponting, 1997; Taylor and Zhulin, 1999; Henry and Crosson, 2011). We speculate that transcriptional activation of *adhA1* and *adhA2* by AdhR is controlled by these sensory modules rather than AdhR abundances. Instead, the presence of a large amount of inactive AdhR protein may be a burden to cells. This may explain why overexpression of AdhR resulted in impaired cell growth and did not enhance solvent production. The studies on the mechanism of transcriptional activation by AdhR, including the signals perceived by the *N*‐terminal regulatory domains and how these domains control the activity of the central AAA^+^ domain, are now under way. A deep insight into the mechanism for AdhR regulation may allow designing sophisticated strategies of AdhR protein engineering to improve solvent production by *C. beijerinckii*.

A previous report has shown that *adhA1* and *adhA2* are members of Rex regulon in *C. beijerinckii* (Zhang *et al.*, 2014). However, inactivation of *rex* had only a modest effect on expression of *adhA1* and *adhA2* and did not change the fermentation product profile in *C. beijerinckii*. Our study indicates that SigL and AdhR are required for transcription of *adhA1* and *adhA2*. Compared with the wild type, ethanol production and butanol production were drastically reduced in *sigL* and *adhR* mutants. This may lead to an increase in intracellular NADH/NAD^+^ ratio and thus depression of Rex target operons including those involved in butyrate synthesis (*crt*‐*bcd*‐*etfBA*‐*hbd* and *ptb*‐*buk* operons). Consistently, we observed markedly increased butyrate synthesis in both *sigL* and *adhR* mutants, which can consume large amounts of NADH. The regulatory system composed of SigL and AdhR and the Rex regulator may sense different signals and act cooperatively to maintain redox homeostasis in *C. beijerinckii*.

This study provided experimental evidence that SigL binds upstream of *adhA* genes in other solvent‐producing species including *C. saccharobutylicum* and *C. saccharoperbutylacetonicum*, suggesting that SigL may also regulate solvent synthesis in these species. We also observed binding of SigL to the promoter regions of ADH‐encoding genes in gas‐fermenting species including *C. ljungdahlii* and *C. autoethanogenum*. Of these *adhA* and *adhP* genes, CAETHG_0386 is involved in 2,3‐butanediol synthesis (Kopke *et al.*, 2011), and CLJU_c24880 and CLJU_c39950 are involved in butanol degradation (Tan *et al.*, 2014). Thus, SigL may play a role in regulation of alcohol metabolism in various *Clostridium* species.

## Experimental procedures

### Strains and culture conditions

The bacterial strains and plasmids used in this study were summarized in Table S1. *Clostridium beijerinckii* NCIMB 8052 strains were grown anaerobically in clostridial growth medium (CGM) (Wiesenborn *et al.*, 1989). Erythromycin (30 μg ml^−1^) or spectinomycin (350 μg ml^−1^) was added when needed. A single colony was inoculated into a test tube containing 5 ml CGM, anaerobically cultured at 37°C for 12 h. Cells were transferred to 50 ml fresh CGM and grown anaerobically to mid‐exponential growth phase (optical density at 600 nm [OD_600_] of ~ 0.6). Then, 2.5 ml of the culture aliquot was transferred to a flask with 60 ml of P2 minimal medium, which contains (per litre) 0.5 g KH_2_PO_4_, 0.5 g K_2_HPO_4_, 0.01 g NaCl, 0.2 g MgSO_4_ · 7H_2_O, 0.01 g MnSO_4_ · H_2_O, 0.01 g FeSO_4_ · 7H_2_O, 1 mg *p*‐aminobenzoic acid, 1 mg vitamin B_1_, 0.01 mg biotin, 2.2 g CH_3_COONH_4_ and 60 g glucose (Baer *et al.*, 1987). The culture was grown anaerobically at 37°C, and samples were taken from the flasks for measurements of OD_600_ and cellular metabolites, RNA isolation and β‐galactosidase assay.

### Mutant construction

Gene disruption in *C. beijerinckii* was performed by using group II intron‐based targetron technology as described previously (Shao *et al.*, 2007). Briefly, a 350 bp fragment for retargeting an intron to insert within the *sigL* (Cbei_0595), *adhR* (Cbei_2180) or Cbei_0091 genes was generated by one‐step assembly PCR using the primers shown in Table S2 based on the protocol of TargeTron^TM^ gene knockout system (Sigma, Sigma‐Aldrich, Darmstadt, Germany). The PCR product was ligated to a targetron vector pWJ1 (Xiao *et al.*, 2011) using One Step Seamless Assembly Super Kit (Paisiwen Co., Ltd, Shanghai, China), yielding the plasmids pWJ1‐sigL, pWJ1‐adhR and pWJ1‐Cbei0091 (Table S1). Each plasmid was electroporated into *C. beijerinckii*. The transformants were selected on CGM plate supplemented with erythromycin. The resulting mutant with an intron insertion in the respective gene was confirmed by PCR.

For complementation of the gene inactivation, the *sigL* and *adhR* genes were PCR‐amplified from the *C. beijerinckii* NCIMB 8052 genomic DNA and cloned into the pXY1 vector under the control of the constitutive *P*_thl_ promoter (Tummala *et al.*, 1999). The obtained plasmids pXY1‐sigL and pXY1‐adhR were electroporated into the *sigL*‐inactivated mutant and the *adhR*‐inactivated mutant respectively (Table S1). For overexpression of *sigL* or *adhR* genes*,* plasmid pXY1‐sigL or pXY1‐adhR was electroporated into wild‐type *C. beijerinckii* (Table S1).

### RNA isolation and real‐time PCR analysis

Total RNA was isolated from *C. beijerinckii* cells that were harvested at OD_600_ of ~ 2.0. RNA isolation, and real‐time PCR analysis was performed as described previously (Zhang *et al.*, 2014).

### β‐Galactosidase assay

For *lacZ* reporter experiments, plasmids containing either a wild‐type or a mutated promoter DNA of *adhA* genes were constructed (Table S1). A 380 bp fragment of *adhA1* (Cbei_2181) promoter and a 475 bp fragment of *adhA2* (Cbei_1722) promoter, both of which contain the putative ‐12 and ‐24 elements and three UAS sites, were PCR‐amplified using *C. beijerinckii* genome as template and the primers shown in Table S2. The mutated promoter DNAs were synthesized by GenScript. The wild‐type or mutated promoter DNA was cloned into pIMPI‐lacZ plasmid to control *lacZ* expression (Feustel *et al.*, 2004). *Clostridium beijerinckii* strains harbouring either the *adhA1* or *adhA2* promoters upstream of the *lacZ* reporter were grown in P2 minimal medium and harvested at OD_600_ of about 2.0 by centrifugation. The crude cell extract was prepared, and the β‐galactosidase activity was determined by measuring the absorbance at 420 nm at 60°C (Tummala *et al.*, 1999). One unit is defined as the amount of the enzyme that catalyzes the formation of 1 nmol of *o*‐nitrophenol (extinction coefficient, 0.0045 μM^−1^ cm^−1^) per min.

### Protein purification

The *sigL* gene and the DNA coding for truncated AdhR (AdhR‐DBD) were PCR‐amplified from *C. beijerinckii* genome using the primers shown in Table S2. The PCR fragments were cloned into the expression vector pET28a (Table S1). The resulting plasmids pET28a‐sigL and pET28a‐adhR‐DBD (Table S1) were sequenced to exclude unwanted mutations and used to produce the N‐terminal hexahistidine‐tagged SigL and AdhR‐DBD respectively. Recombinant protein overexpression and purification were performed as described previously (Nie *et al.*, 2016).

### Electrophoretic mobility shift assay

A 200 bp DNA fragment containing the putative −24 and −12 elements in the promoter region of *adhA1* or *adhA2* genes was PCR‐amplified from *C. beijerinckii* genome using the primers shown in Table S2. The DNA fragments containing the putative −24 and −12 elements upstream of CLSA_RS07875 gene from *C. saccharobutylicum*, Cspa_c27830 gene from *C. saccharoperbutylacetonicum*, CLJU_c24880 and CLJU_c39950 genes from *C. ljungdahlii* and CAETHG_0385 gene from *C. autoethanogenum* were chemically synthesized by GenScript. The DNA fragment containing the three UAS sites in the promoter region of *adhA1* or *adhA2* genes was PCR‐amplified from *C. beijerinckii* genome using the primers shown in Table S2. Both forward and reverse primers were Cy5 fluorescence labelled at the 5′‐end. Mobility shift assays were performed as described previously (Zhang *et al.*, 2014).

### Metabolite analysis

For analysis of extracellular metabolites, culture samples were centrifuged for 10 min at 4°C and 15 000 *g* to remove the cells. Acetone, butanol and ethanol were detected by gas chromatography as described previously (Liu *et al.*, 2012). Glucose concentration in culture supernatant was detected by high‐pressure liquid chromatography using an Agilent model 1260 instrument equipped with a Sugar‐Pak^TM^ I column (Waters, Milford, MA, USA) and a refractive index detector (Agilent Technologies, Santa Clara, CA, USA).

### Statistical analysis

Unless noted otherwise, data are presented as the mean ± SD of *n* independent experiments.

## Conflict of interests

None declared.

## Supporting information


**Fig. S1**. Construction and validation of gene inactivation mutants of *C. beijerinckii*. The coding region of *sigL* (Cbei_0595) or *adhR* (Cbei_2180) genes was inserted with an intron. The resulting mutants were confirmed by PCR.
**Table S1**. Strains and plasmids used in this study.
**Table S2**. Oligonucleotides used in this study.
