# Supplementary material for: Control of solvent production by sigma‐54 factor and the transcriptional activator AdhR in Clostridium beijerinckii
Source: Microb Biotechnol. 2019 Nov 6;13(2):328–38. doi: 10.1111/1751-7915.13505 (PMC7017808; doi:10.1111/1751-7915.13505)
Supplement: Supplementary file 1 — Fig. S1. Construction and validation of gene inactivation mutants of C. beijerinckii. The coding region of sigL (Cbei_0595) or adhR (Cbei_2180) genes was inserted with an intron. The resulting mutants were confirmed by PCR. Table S1. Strains and plasmids used in this study. Table S2. Oligonucleotides used in this study. [file MBT2-13-328-s001.docx]

**Supporting Information**

**Control of solvent production by sigma-54 factor and the transcriptional activator AdhR in *Clostridium beijerinckii***

Bin Yang, Xiaoqun Nie, Yang Gu, Weihong Jiang, Chen Yang^*^

^*^For correspondence: E-mail [chenyang@sibs.ac.cn](mailto:chenyang@sibs.ac.cn).

**Fig. S1** Construction and validation of gene inactivation mutants of *C. beijerinckii*. The coding region of *sigL* (Cbei_0595) or *adhR* (Cbei_2180) genes was inserted with an intron. The resulting mutants were confirmed by PCR.

| Strains | Genotype | Source |
| --- | --- | --- |
| *C. beijerinckii* strains | | |
| 8052 | Wild type | NCMIB |
| *sigL* mutant | *sigL*::intron | This work |
| *adhR* mutant | *adhR*::intron | This work |
| Cbei_0091 mutant | Cbei_0091::intron | This work |
| *sigL* comp | *sigL*::intron/pXY1-sigL | This work |
| *adhR* comp | *adhR*::intron/pXY1-adhR | This work |
| pXY1 | Wild type/pXY1 | This work |
| pXY1-sigL | Wild type/pXY1-sigL | This work |
| Plasmids |  |  |
| pWJ1 | Em^r^; Amp^r^; pcb102; *P*_ptb_; Group II intron | (Xiao, et al., 2011) |
| pXY1 | Amp^r^; Spec^r^; pcb102, ColE1 origin | (Sun, et al., 2015) |
| pIMP1-lacZ | Amp^r^; Em^r^; repL; *lacZ* | (Feustel, et al., 2004) |
| pWJ1-sigL | Amp^r^; Em^r^; pcb102; *P*_ptb_; inserting intron into *sigL* | This work |
| pWJ1-adhR | Amp^r^; Em^r^; pcb102; *P*_ptb_; inserting intron into *adhR* | This work |
| pWJ1-Cbei0091 | Amp^r^; Em^r^; pcb102; *P*_ptb_; inserting intron into Cbei_0091 | This work |
| pXY1-sigL | Amp^r^; Spec^r^; pcb102; *P_thl_ sigL* | This work |
| pXY1-adhR | Amp^r^; Spec^r^; pcb102; *P_thl_ adhR* | This work |
| pRS-lacZ | Amp^r^; Em^r^; pcb102; Derived from pIMP1-lacZ | This work |
| pPadhA-lacZ1 | Amp^r^; Em^r^; pcb102; *P_adhA1_* *lacZ* | This work |
| pPadhA-lacZ2 | Amp^r^; Em^r^; pcb102; *P_adhA1_*_-M24_ *lacZ* | This work |
| pPadhA-lacZ3 | Amp^r^; Em^r^; pcb102; *P_adhA1_*_-M12_ *lacZ* | This work |
| pPadhA-lacZ4 | Amp^r^; Em^r^; pcb102; *P_adhA1_*_-M1_ *lacZ* | This work |
| pPadhA-lacZ5 | Amp^r^; Em^r^; pcb102; *P_adhA1_*_-M2_ *lacZ* | This work |
| pPadhA-lacZ6 | Amp^r^; Em^r^; pcb102; *P_adhA1_*_-M3_ *lacZ* | This work |
| pPadhA-lacZ7 | Amp^r^; Em^r^; pcb102; *P_adhA1_*_-M4_ *lacZ* | This work |
| pPadhA-lacZ8 | Amp^r^; Em^r^; pcb102; *P_adhA2_ lacZ* | This work |
| pPadhA-lacZ9 | Amp^r^; Em^r^; pcb102; *P_adhA2_*_-M24_ *lacZ* | This work |
| pPadhA-lacZ10 | Amp^r^; Em^r^; pcb102; *P_adhA2_*_-M12_ *lacZ* | This work |
| pPadhA-lacZ11 | Amp^r^; Em^r^; pcb102; *P_adhA2_*_-M1_ *lacZ* | This work |
| pPadhA-lacZ12 | Amp^r^; Em^r^; pcb102; *P_adhA2_*_-M2_ *lacZ* | This work |
| pPadhA-lacZ13 | Amp^r^; Em^r^; pcb102; *P_adhA2_*_-M3_ *lacZ* | This work |
| pPadhA-lacZ14 | Amp^r^; Em^r^; pcb102; *P_adhA2_*_-M4_ *lacZ* | This work |
| pET28a | Kan^r^; poly-His tag; *P*_T7_ | Novagen |
| pET28a-sigL | Kan^r^; *P*_T7_ *sigL* | This work |
| pET28a-adhR-DBD | Kan^r^; *P*_T7_ adhR-DBD | This work |

**Table S1** Strains and plasmids used in this study.

**Table S2** Oligonucleotides used in this study.

| Name | Sequence 5ʹ → 3ʹ | Used for plasmid |
| --- | --- | --- |
| Primer1 | AAAAAAGCTTATAATTATCCTTAGACATCGATCAAGTGCGCCCAGATAGGGTG | pWJ1-sigL |
| Primer2 | CAGATTGTACAAATGTGGTGATAACAGATAAGTCGATCAATATAACTTACCTTTCTTTGT | pWJ1-sigL |
| Primer3 | TGAACGCAAGTTTCTAATTTCGATTATGTCTCGATAGAGGAAAGTGTCT | pWJ1-sigL |
| Primer4 | CGAAATTAGAAACTTGCGTTCAGTAAAC | pWJ1-sigL, pWJ1-adhR, pWJ1-Cbei0091 |
| Primer5 | AAAAAAGCTTATAATTATCCTTAGCCATCGTTAATGTGCGCCCAGATAGGGTG | pWJ1-adhR |
| Primer6 | AAAAAAGCTTATAATTATCCTTAGCCATCGTTAATGTGCGCCCAGATAGGGTG | pWJ1-adhR |
| Primer7 | TGAACGCAAGTTTCTAATTTCGATTATGGCTCGATAGAGGAAAGTGTCT | pWJ1-adhR |
| Primer8 | AAAACTCGAGATAATTATCCTTATTTCTCCTTTTTGTGCGCCCAGATAGGGTG | pWJ1-Cbei0091 |
| Primer9 | CAGATTGTACAAATGTGGTGATAACAGATAAGTCCTTTTTCTTAACTTACCTTTCTTTGT | pWJ1-Cbei0091 |
| Primer10 | TGAACGCAAGTTTCTAATTTCGGTTAGAAATCGATAGAGGAAAGTGTCT | pWJ1-Cbei0091 |
| Primer11 | GGAGTGTCGAGGATCCCCGGGATGAATTTGGATTATAACATG | pXY1-sigL |
| Primer12 | GAAAATACCGCATCAGGCGCCTTATAATCTCTTCCTTGCAG | pXY1-sigL |
| Primer13 | GTAAAAGGGAGTGTCGAGGATCCATGGAGAATAAAGAACTATTAATTG | pXY1-adhR |
| Primer14 | CTGAGAGTGCACCATATGTCGACTTATTTATGTTTTTTTAATTTTAGG | pXY1-adhR, |
| Primer15 | AGAGAGCTGCAGATTATAAAGCATGTCCTAAA | pPadhAlacZ1 |
| Primer16 | AGAGAGGGATCCAAATATTTCCTCCTAAAATTTG | pPadhAlacZ1 |
| Primer17 | AGAGAGCTGCAGAGGATTATTTTTAGTGGC | pPadhAlacZ8 |
| Primer18 | AGAGAGGGATCCAAATTTATACCTCCTAAAA | pPadhAlacZ8 |
| Primer19 | AGAGAGGGATCCATGAATTTGGATTATAACATG | pET28a-sigL |
| Primer20 | AGAGAGGCGGCCGCTTATAATCTCTTCCTTGCAG | pET28a-sigL |
| Primer21 | AGAGAGGGATCCGATGGACGATCATCTTTTAATTTATGGG | pET28a-adhR-DBD |
| Primer22 | AGAGAGGCGGCCGCTTATTTATGTTTTTTTAATTTTAGG | pET28a-adhR-DBD |
| Primer23 | TGAGGAAATAAGCTCATATGATGATG | Confirmation of sigL disruption |
| Primer24 | ATCTTTTCTATTACATCATCTAAAAT | Confirmation of sigL disruption |
| Primer25 | ATATTATAAAGGAATTAAAATTAAAG | Confirmation of adhR disruption |
| Primer26 | GTCCCATCCTCATACCCAAATAATTC | Confirmation of adhR disruption |
| Primer27 | GTGAATAAAGCAGAATTAATTAC | Confirmation of Cbei_0091 disruption |
| Primer28 | TTACTTGTTAACTTTATCTTTG | Confirmation of Cbei_0091 disruption |
| Primer29 | AGCCAGTGGCGATAAGAAAGTAAAACACATAATAATTG | PadhA1-SigL EMSA |
| Primer30 | AGCCAGTGGCGATAAGTTCGCTCTCTCCTTTTAATA | PadhA1-SigL EMSA |
| Primer31 | AGCCAGTGGCGATAAGGCAGTGTGTAAGTTAAATTAC | PadhA2-SigL EMSA |
| Primer32 | AGCCAGTGGCGATAAGTTTTAACCTCTCCTTGGATA | PadhA2-SigL EMSA |
| Primer33 | AGCCAGTGGCGATAAGATTATAAAGCATGTCCTA | PadhA1-DBD EMSA |
| Primer34 | AGCCAGTGGCGATAAGAACAAAAAACTCGTAATTC | PadhA1-DBD EMSA |
| Primer35 | AGCCAGTGGCGATAAGTTAAATAAATAAACTATC | PadhA2-DBD EMSA |
| Primer36 | AGCCAGTGGCGATAAGTCTCCCCATTTATTGAAAT | PadhA2-DBD EMSA |
| Primer33 | GAAGAATACCAGTGGCGAAGGC | qRT-PCR Internal control |
| Primer34 | ATTCATCGTTTACGGCGTGGAC | qRT-PCR Internal control |
| Primer35 | GAGATATTGCGAGAGCCTTA | qRT-PCR adhA1 |
| Primer36 | ATACTGCGTTATGAGCGATA | qRT-PCR adhA1 |
| Primer37 | CTGATTGGATAGTTGCTA | qRT-PCR adhA2 |
| Primer38 | CACTTGTTGATGGAATAG | qRT-PCR adhA2 |

**References**

Feustel, L., Nakotte, S., and Durre, P. (2004) Characterization and development of two reporter gene systems for Clostridium acetobutylicum. *Appl Environ Microbiol* **70**: 798-803.

Sun, Z., Chen, Y., Yang, C., Yang, S., Gu, Y., and Jiang, W. (2015) A novel three-component system-based regulatory model for D-xylose sensing and transport in Clostridium beijerinckii. *Mol Microbiol* **95**: 576-589.

Xiao, H., Gu, Y., Ning, Y., Yang, Y., Mitchell, W.J., Jiang, W., and Yang, S. (2011) Confirmation and elimination of xylose metabolism bottlenecks in glucose phosphoenolpyruvate-dependent phosphotransferase system-deficient Clostridium acetobutylicum for simultaneous utilization of glucose, xylose, and arabinose. *Appl Environ Microbiol* **77**: 7886-7895.
